# Impact of segmental body composition on metabolic *dysfunction-associated* fatty liver disease in Chinese children

**DOI:** 10.3389/fendo.2025.1505050

**Published:** 2025-02-17

**Authors:** Meng-Yuan Hu, Dan-Qin Sun, Fan Yang, Xiao-Wei Zheng, Ning-Xi Wu, Hao-Yang Zhang, Xiao-Die Yao, Jia-Hui Zhang, Le Zhang

**Affiliations:** ^1^ Department of Paediatrics, Affiliated Children’s Hospital of Jiangnan University, Wuxi Children’s Hospital, Wuxi, Jiangsu, China; ^2^ Urologic Nephrology Center, Jiangnan University Medical Center, Wuxi, Jiangsu, China; ^3^ Wuxi School of Medicine, Jiangnan University, Wuxi, Jiangsu, China

**Keywords:** BIA, MAFLD, children, regional body composition, Fibroscan

## Abstract

**Purpose:**

This study aimed to assess the relationship between regional body composition and metabolic dysfunction-associated fatty liver disease (MAFLD) in Chinese children.

**Methods:**

In this study, 1399 children aged 7–14 years were included. Liver steatosis was assessed using the controlled attenuation parameter (CAP) measured through Fibroscan. MAFLD is defined as the presence of liver steatosis along with either overweight/obesity, prediabetes/diabetes, or at least two metabolic index abnormalities. Regression analyses were applied to assess the relationship between regional body composition and MAFLD in children. Subgroup analyses were performed based on sex and weight.

**Results:**

The participants had a mean age of 9 years, with 52.11% being boys. Among them, 134 (9.57%) were diagnosed with MAFLD, and 17 (1.22%) had severe fatty liver disease. We found an inverse correlation between the muscle percentage in each region and MAFLD, with the extremities demonstrating the most significant negative correlation (OR: 0.732; 95% CI: 0.634–0.844). Conversely, regional fat was positively associated with MAFLD, with the strongest correlation found in the upper limbs (OR: 3.104; 95% CI: 2.023–4.764). Subgroup analyses showed similar results.

**Conclusion:**

The decrease in regional muscle percentage, particularly in the limbs, along with the increase in regional fat percentage, especially in the upper limbs, is associated with a higher probability of developing MAFLD in prepubertal children. Additional prospective studies are needed to strengthen and validate these findings.

## 1 Introduction

In recent years, the global prevalence of childhood obesity has surged, emerging as a significant public health challenge. It not only contributes to short-term health issues, such as metabolic disorders and psychological problems, but also markedly increases the risk of chronic diseases in adulthood, including metabolic syndrome, diabetes, and cardiovascular diseases. Childhood obesity is closely associated with metabolic-associated fatty liver disease (MAFLD), which is the leading cause of chronic liver disease in children (1). The estimated prevalence of MAFLD in children is approximately 10% in the general population and up to 34% in children who are obese (2). Moreover, the prevalence of MAFLD varies across different regions. In obese children, MAFLD prevalence is higher in Asia (52.1%) than in Europe (39.7%) and North America (23.0%) (3). Pediatric MAFLD is associated with hepatic and extrahepatic comorbidities, including hypertension, dyslipidemia, gallstones, diabetes, chronic kidney disease, and depression (4–6). Research has shown that pediatric fatty liver disease may persist into adulthood and significantly reduce life expectancy (7).

Recently, there has been growing interest in assessing body composition in MAFLD patients. Epidemiological studies indicate that low skeletal muscle mass is linked to a higher likelihood of developing MAFLD and its severity in adults (8–11). Guo et al. reported that participants in the lowest tertiles of skeletal muscle mass had an approximately fourfold higher risk of developing MAFLD and an approximately fourfold higher risk of advanced fibrosis compared to those in the highest tertile (9). A possible explanation is that skeletal muscle mass affects liver metabolic health by modulating insulin-mediated glucose disposal, which plays a critical role in the pathogenesis of MAFLD (12). In addition, increased body fat mass, especially abdominal fat deposition, is linked to a higher incidence of MAFLD (13–15). A large observational study predicted that for every 1 kg increase in fat mass, participants had a 27%–40% increased risk of MAFLD (13). The researchers also confirmed a correlation between muscle and fat mass in adults and MAFLD risk.

Despite the abundant data linking body composition to MAFLD in adults, research in pediatric populations remains limited. Therefore, the study was designed to assess muscle and fat content in various body regions and explore their relationship with MAFLD in children using bioelectrical impedance analysis (BIA).

## 2 Methods

### 2.1 Study design and participants

**Figure 1:**
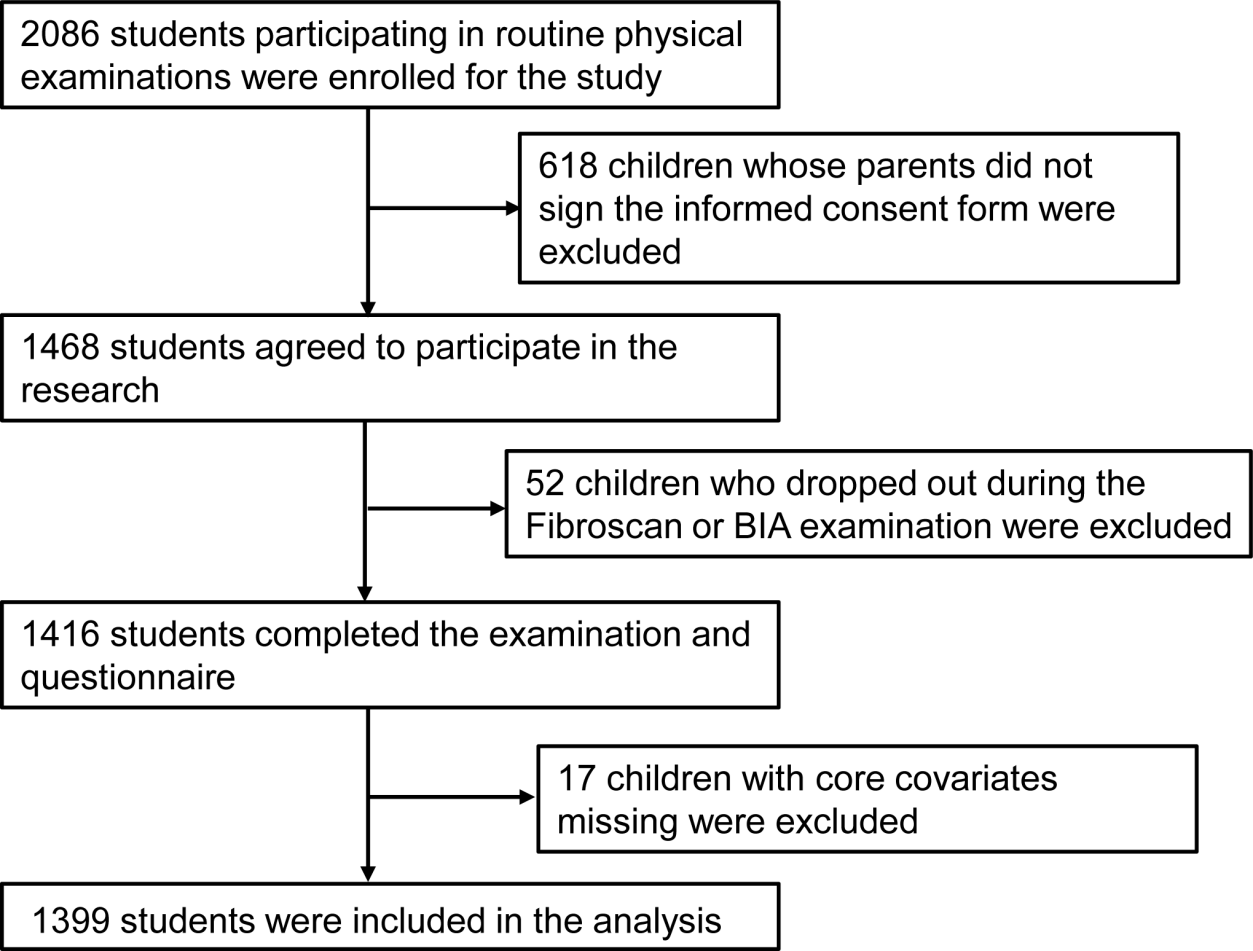
Flowchart of participants selection.

This cohort study was conducted in Wuxi City, Jiangsu Province, China, and involved approximately 2086 students aged 7 to 14 years, from March to April 2023. Initially, these students were required to undergo the routine elementary school physical examination, and participants were recruited from this group. Subsequently, 687 children were excluded for the following reasons: (1) 618 children had parents who did not sign the informed consent form; (2) 52 children dropped out during the Fibroscan and/or BIA examination; and (3) 17 children were excluded because of incomplete core data. Ultimately, a total of 1399 children were enrolled in this study (**Figure 1**). Personal information was omitted, and personal identifiers were replaced with health examination numbers. This study was approved by the Wuxi Children’s Hospital, Affiliated Hospital of Jiangnan University. Written informed consent was obtained from the guardians of the participants before their participation in the study.

### 2.2 Anthropometric measurements and body composition evaluation

Standard procedures were followed by trained technicians to obtain anthropometric data. After an overnight fast, all participants were asked to wear light clothing for BIA measurements. BIA was conducted using a fixed multifrequency, eight-point device (InBody 370, InBody, Seoul, Korea). Participants stood on the scale’s footpads (with two electrodes per foot) and held a handle in each hand (with two electrodes per hand) for approximately 1 minute. The device, which measured fat and muscle mass for each region (bilateral upper and lower limbs and trunk), recorded height, sex and age. Muscle mass percentage was defined as the ratio of muscle mass to body weight, calculated as [muscle mass (kg)/weight (kg)] × 100 (16). Percentage of regional muscle mass, e.g. upper extremity, was calculated by dividing sum of left and right upper extremity muscle mass by body weight. The following formula was used to calculate body fat percentage: [fat mass (kg)/weight (kg)] × 100. For assessing regional body fat percentage, we used the following formula: [regional body fat mass (kg)/weight (kg)] × 100. The lower limb fat mass was calculated by adding the fat mass on both sides of the lower limbs, and the same method was applied for other regions.

### 2.3 Clinical and biochemical measurements

The data were collected by trained medical staff at the school. General examinations included measurements of height, weight, and blood pressure (BP). Weight was measured in kilograms, height in centimeters, and BP in millimeters of mercury (mmHg). Venous samples were collected after an 8-hour overnight fast. Serum levels of alanine aminotransferase (ALT), gamma-glutamyl transferase (GGT), aspartate aminotransferase (AST), total cholesterol (TC), triglyceride (TG), fasting glucose, high-density lipoprotein (HDL) cholesterol, and low-density lipoprotein (LDL) cholesterol were measured. The homeostasis model assessment of insulin resistance (HOMA-IR) score (17) was calculated as follows: HOMA-IR = fasting insulin [µIU/mL] × fasting plasma glucose [mmol/L])/22.5.

### 2.4 Liver steatosis and MAFLD definition

Transient elastography using the Fibroscan device (Echosens, Paris, France) was conducted by operators using a 3.5 MHz M probe. All operators were trained according to a standardized procedure and had each conducted more than 50 examinations prior to the study. Ten successful measurements were performed to obtain the average controlled attenuation parameter (CAP) scores. According to the user manual, participants were classified based on the CAP value into the following groups: non-liver steatosis (CAP < 238 dB/m), mild liver steatosis (238 dB/m ≤ CAP value < 259 dB/m), moderate liver steatosis (259 dB/m ≤ CAP value < 292 dB/m), or severe liver steatosis (CAP value ≥ 292 dB/m) groups (18). In line with the most recent consensus, the diagnosis of MAFLD in children includes the presence of steatosis, assessed using the CAP value, along with at least one of the following criteria: overweight/obesity, prediabetes, or diabetes, and at least two metabolic abnormalities (19). Overweight and obesity were defined based on the BMI z-score, according to the age and sex-specific criteria proposed by the World Health Organization (20). Abdominal obesity was diagnosed when waist circumference exceeded the 90th percentile for age and gender (21). Prediabetes and diabetes were defined according to international guidelines (22). Diagnostic criteria included: (1) a previous diagnosis of diabetes; (2) a hemoglobin A1c level of 5.7% (48 mmol/mol) or higher; and (3) a fasting plasma glucose level of 100 mg/dL or higher. These metabolic abnormalities were accompanied by elevated BP, elevated TG levels, low high-density lipoprotein levels, and high TG/HDL ratios (23). Elevated BP was defined as systolic or diastolic BP exceeding the 90th percentile, while elevated TG levels were defined as TG levels above the 90th percentile. A low HDL level was defined as an HDL level ≤ 10th percentile. The diagnostic criteria for children aged 10–15 years were as follows: elevated BP (defined as a systolic reading > 130 mmHg or diastolic reading > 85 mmHg); TG levels ≥ 150 mg/dL; HDL cholesterol levels < 40 mg/dL; and a TG-to-HDL cholesterol ratio > 2.25 (19).

### 2.5 Questionnaire

Parents completed a questionnaire on their children’s lifestyles. The questionnaire gathered basic information regarding the children’s screen time and physical activity status. Screen time was categorized as < 30 min/day, 1–2 hour/day, or > 2 hours/day. For physical activity, exercise habits were classified as yes or no; exercise frequency was classified as seldom, 1–3 times/week, 4–6 times/week or every day; exercise duration was classified as < 30 min, 30–90 min or 90–120 min. Based on the examination results, valid questionnaires were grouped into MAFLD and non-MAFLD categories. The relationship between pediatric MAFLD and lifestyle habits was investigated.

### 2.6 Statistical analysis

Continuous variables are presented as mean ± standard deviation. Statistical comparisons across groups were conducted using either Student’s t-test or one-way analysis of variance. Categorical variables expressed as percentages, were compared using the chi-squared test. The logistic regression model was used to evaluate the associations between regional muscle mass percentage, regional fat mass percentage, and the risk of MAFLD. Three models were established, and adjustments were made for various factors. These factors included age, sex, prediabetes or diabetes status, overweight status, central obesity status, elevated BP, elevated TG, low HDL, and a high TG/HDL ratio. Stratified analyses were conducted based on sex (male and female) and weight (normal weight or overweight/obesity). All statistical analyses were performed using SPSS (version 26.0). The significance level was set at two-side *P* < 0.05.

## 3 Results

### 3.1 Participant characteristics

**Figure 2:**
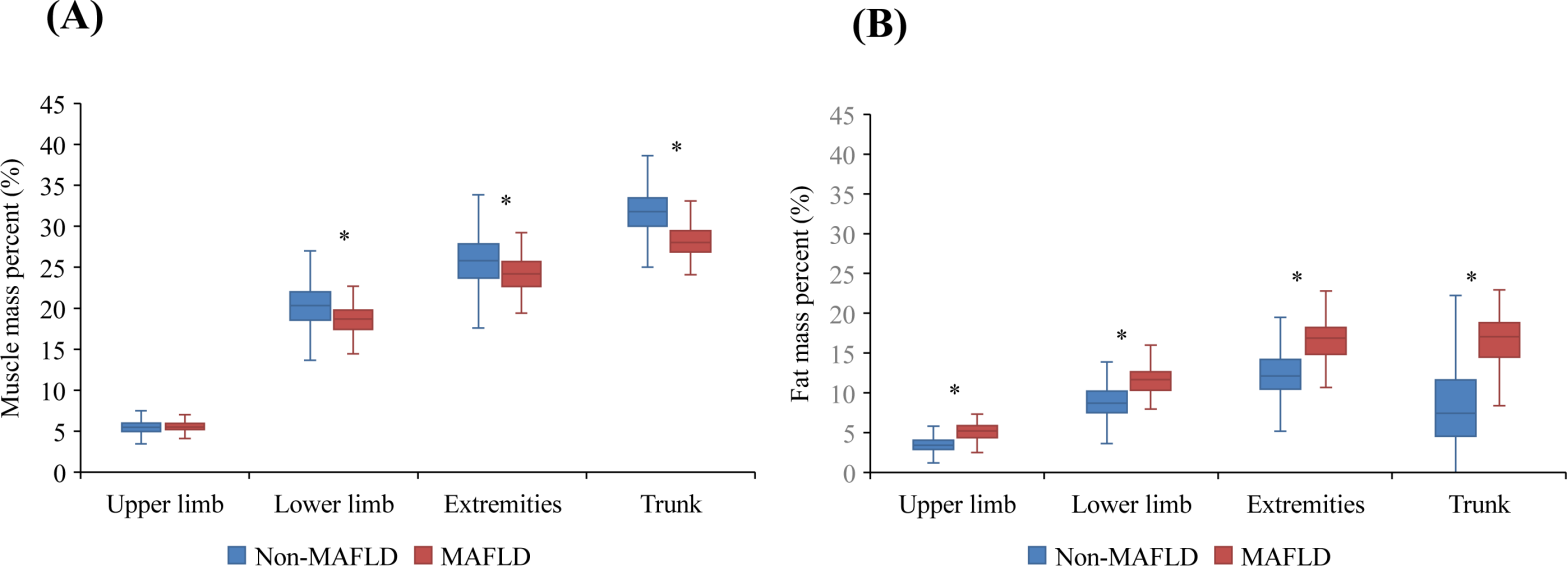
Regional muscle mass percentage (%)/fat mass percentage (%) between the MAFLD and non-MAFLD groups. **(A)** Regional muscle mass percentage (%) between the MAFLD and non-MAFLD groups. **(B)** Regional fat mass percentage (%) between the MAFLD and non-MAFLD groups. * indicate *P* < 0.05.

As shown in **Table 1**, the study included 1,399 children with a mean age of 9 ± 2 years. Of these, 52.11% were boys and the mean BMI z score was 0.58 ± 1.19. Fatty liver disease was observed in 178 participants (12.71%), with 17 (1.22%) having severe fatty liver disease. Among these patients, 134 (9.57%) were diagnosed with MAFLD. Compared to the non-MAFLD group, they were more likely to have significantly greater systolic and diastolic BP, decreased HDL cholesterol levels, increased TG levels, and a greater prevalence of prediabetes or diabetes. They also had significantly greater BMI, waist circumferences, TG levels, fasting plasma glucose levels, ALT levels, GGT levels, HOMA-IR scores, and lower HDL cholesterol levels than those in the non-MAFLD group (*P* < 0.05). In terms of body composition parameters, patients with MAFLD had significantly lower total muscle mass percentage (34.34 ± 2.91% *vs.* 39.34 ± 3.75%, *P* < 0.05) and greater total body fat percentage (34.53 ± 6.92% *vs.* 22.57 ± 7.06%, *P* < 0.05) than patients in the non-MAFLD group. As shown in **Figure 2**, compared to the non-MAFLD group, the MAFLD group had significantly lower muscle mass percentages in the lower limbs (18.67 ± 1.80% *vs.* 20.40 ± 2.64%, *P* < 0.05), extremities (24.23 ± 2.20% *vs.* 25.9 ± 3.18%, *P* < 0.05), and trunk (28.22 ± 1.96% *vs.* 31.74 ± 2.55%, *P* < 0.05). Furthermore, the fat mass percentages in the upper limbs (5.13 ± 1.08% *vs.* 3.53 ± 0.90%, *P* < 0.05), lower limbs (11.65 ± 1.66% *vs.* 8.90 ± 1.99%, *P* < 0.05), extremities (16.78 ± 2.63% *vs.* 12.44 ± 2.83%, *P* < 0.05), and trunk (16.32 ± 4.05% *vs.* 8.21 ± 4.77%, *P* < 0.05) were significantly greater in the MAFLD group than in the non-MAFLD group.

**Table 1:** Baseline characteristics of participants.

Variables	All (N = 1399)	Non-MAFLD (N = 1265)	MAFLD (N = 134)	*P*
Age (years)	9 ± 2	9 ± 2	10 ± 2	**< 0.001**
Sex, male, n (%)	729 (52.11)	627 (49.57)	102 (76.12)	**< 0.001**
BMI z-score	0.58 ± 1.19	0.41 ± 1.10	2.15 ± 0.78	**< 0.001**
SBP (mm Hg)	107.79 ± 12.3	106.54 ± 11.7	116.9 ± 12.85	**< 0.001**
DBP (mm Hg)	64.12 ± 8.49	63.7 ± 8.43	67.21 ± 8.36	**< 0.001**
HOMA-IR score	1.45 ± 1.05	1.31 ± 0.81	2.62 ± 1.87	**< 0.001**
Waist circumference (cm)	62.41 ± 8.02	61.18 ± 6.67	74.59 ± 9.93	**< 0.001**
Prediabetes or diabetes (%)	19 (1.65)	11 (1.06)	8 (6.90)	**< 0.001**
Overweight or Obesity (%)	498 (35.90)	371 (29.59)	127 (95.49)	**< 0.001**
Central obesity (%)	108 (7.76)	47 (3.74)	61 (45.86)	**< 0.001**
Elevated BP (%)	51 (5.48)	43 (5.26)	8 (7.14)	0.411
Elevated triglycerides (%)	122 (10.36)	100 (9.43)	22 (18.64)	**0.002**
Low HDL (%)	17 (1.48)	11 (1.07)	6 (5.17)	**< 0.001**
High triglyceride/HDL ratio (%)	109 (9.62)	81 (7.96)	28 (24.14)	**< 0.001**
CAP value (dB/m)	193.92 ± 39.11	186.63 ± 32.91	262.71 ± 22.71	**< 0.001**
LSM value (kPa)	4.46 ± 1.06	4.44 ± 1.05	4.7 ± 1.13	**0.006**
ALT (U/L)	14.71 ± 8.69	14.17 ± 7.6	19.76 ± 14.63	**< 0.001**
GGT (U/L)	13.77 ± 3.80	13.40 ± 2.95	17.25 ± 7.48	**< 0.001**
AST (U/L)	25.82 ± 5.89	25.96 ± 5.81	24.43 ± 6.50	**0.013**
TG (mmol/L)	0.75 ± 0.32	0.73 ± 0.30	0.95 ± 0.44	**< 0.001**
TC (mmol/L)	4.72 ± 0.84	4.74 ± 0.84	4.62 ± 0.82	0.110
LDL (mmol/L)	2.28 ± 0.61	2.28 ± 0.60	2.26 ± 0.62	0.735
HDL (mmol/L)	1.67 ± 0.34	1.69 ± 0.34	1.42 ± 0.27	**< 0.001**
FPG (mmol/L)	4.88 ± 0.32	4.86 ± 0.32	5.04 ± 0.33	**< 0.001**
Total muscle mass percent (%)	38.86 ± 3.96	39.34 ± 3.75	34.34 ± 2.91	**< 0.001**
Total body fat percent (%)	23.72 ± 7.82	22.57 ± 7.06	34.53 ± 6.92	**< 0.001**
Daily screen viewing time, n (%)				0.991
< 30 min	1182 (86.72)	1069 (86.80)	113 (85.61)	
1-2 hour	156 (11.40)	138 (11.21)	18 (13.64)	
>2 hours	25 (1.83)	24 (1.95)	1 (0.76)	
Exercise habits, n (%)				0.089
No	148 (10.62)	128 (10.17)	20 (14.90)	
Yes	1245 (89.38)	1131 (89.83)	114 (85.07)	
Exercise frequency, n (%)				0.970
Seldom	153 (11.47)	134 (11.07)	19 (15.32)	
1-3 per week	787 (59.00)	717 (59.26)	70 (56.45)	
4-6 per week	255 (19.12)	239 (19.75)	16 (12.90)	
Everyday	139 (10.42)	120 (9.92)	19 (15.32)	
Exercise duration (min), n (%)				0.071
< 30	474 (35.56)	436 (36.06)	38 (30.65)	
30-90	750 (56.26)	678 (56.08)	72 (58.06)	
90-120	91 (6.83)	81 (6.70)	10 (8.06)	

Continuous variables are shown as mean ± SD. Categorical values are shown as n (%). Bold values indicate if *P* < 0.05. BMI, body mass index; BP, blood pressure; DBP, diastolic blood pressure; HOMA-IR, homeostasis model assessment of insulin resistance; HDL, high-density lipoprotein; MAFLD, metabolic dysfunction–associated fatty liver disease; SBP, systolic blood pressure; PBF (%), percent body fat; CAP, controlled attenuation parameter; LSM, liver stiffness measurement; ALT, alanine aminotransferase; AST, aspartate aminotransferase; GGT, g-glutamyl transferase; TG, triglycerides; TC, total cholesterol; LDL-C, low-density lipoprotein cholesterol; HDL-C, high-density lipoprotein cholesterol; FPG, fasting plasma glucose.

### 3.2 Relationship between segmental body composition and MAFLD

**Figure 3:**
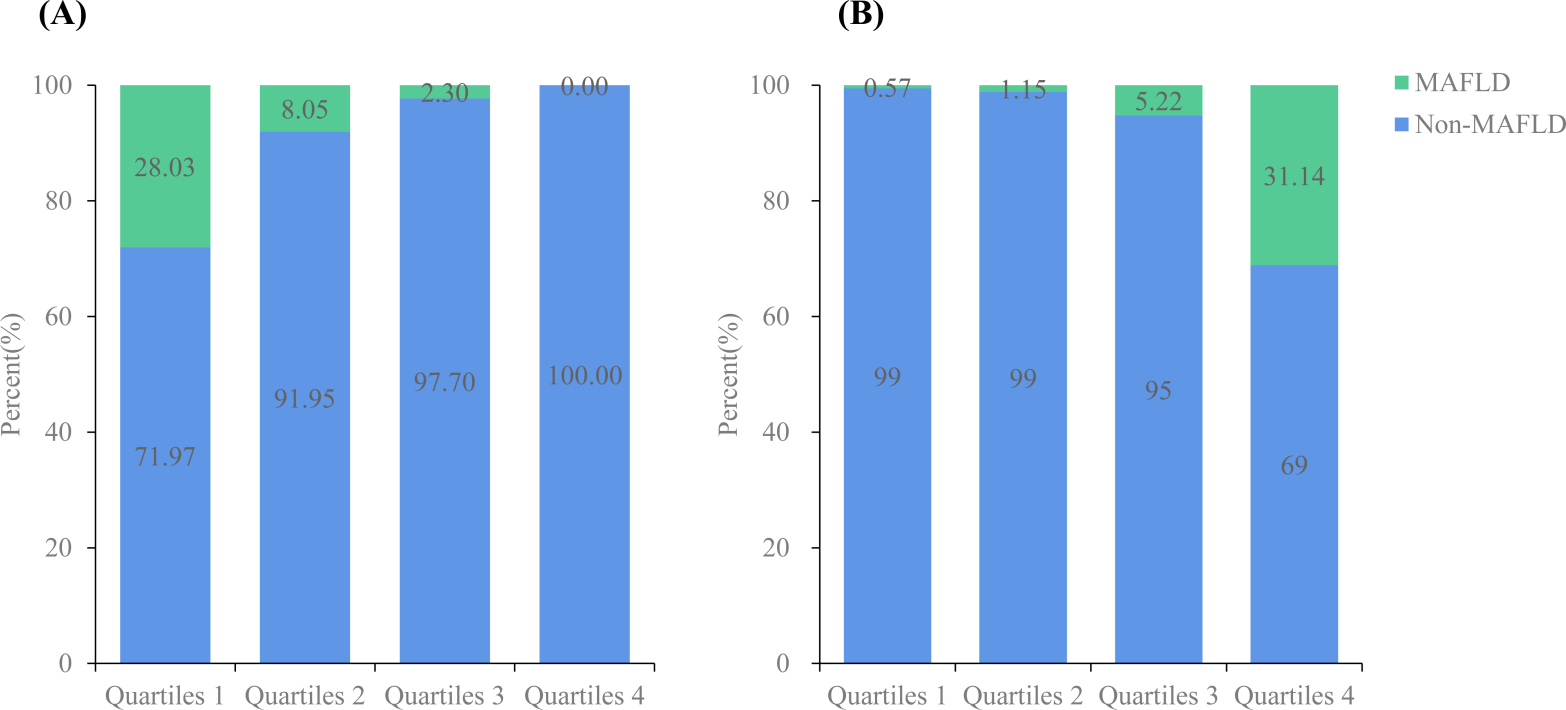
Prevalence of MAFLD according to total muscle mass percentage (%)/fat mass percentage (%). **(A)** The prevalence of non-MAFLD and MAFLD according to total muscle mass percentage (%); **(B)** The prevalence of non-MAFLD and MAFLD according to fat muscle mass percentage (%).

**Figure 3** shows the total muscle and fat mass percentages divided into four quartiles. The prevalence of MAFLD gradually decreased as the muscle mass percentage quartiles increased (28.03% *vs.* 8.05% *vs.* 2.30% *vs.* 0.00%), whereas it increased progressively with higher fat mass percentage quartiles (0.57% *vs.* 1.15% *vs.* 5.22% *vs.* 31.14%).

Logistic regression analysis was conducted to further understand the relationship between MAFLD and segmental body composition (**Table 2**). In the unadjusted model, the muscle mass percentage of each body segment was negatively correlated with MAFLD. In contrast, the risk of MAFLD was significantly associated with the fat mass percentage in each body segment. Importantly, even after adjusting for age, sex, prediabetes or diabetes status, overweight status, central obesity status, elevated BP, elevated TG, low HDL, and a high TG/HDL ratio (Model 3), this relationship remained robust. Furthermore, in Model 3, the percentage of muscle mass in the extremities (OR: 0.732; 95% CI: 0.634–0.844, *P* < 0.001) and lower limbs (OR: 0.682; 95% CI: 0.571–0.813, *P* < 0.001) had a more significant impact on MAFLD than in other body regions. The increased risk of MAFLD was associated with an increase in the percentage of fat mass in the upper limbs (OR: 3.104; 95% CI: 2.023–4.764, *P* < 0.001), lower limbs (OR: 1.794; 95% CI: 1.425–2.257, *P* < 0.001), extremities (OR: 1.499; 95% CI: 1.285–1.749, *P* < 0.001) and trunk (OR: 1.506; 95% CI: 1.299–1.744, *P* < 0.001).

**Table 2:** Multivariate analysis for the relationship between regional muscle mass (%)/fat mass (%) and MAFLD.

Variables	Model 1		Model 2		Model 3	
	OR (95% CI)	*P*	OR (95% CI)	*P*	OR (95% CI)	*P*
Muscle mass percent (%)						
Total	0.695 (0.655-0.738)	**< 0.001**	0.685 (0.642-0.729)	**< 0.001**	0.719 (0.634-0.815)	**< 0.001**
Upper limb	1.125 (0.893-1.418)	0.317	0. 789 (0.611-1.020)	0.070	0.407 (0.241-0.685)	**< 0.001**
Lower limb	0.751 (0.695-0.813)	**< 0.001**	0.592 (0.535-0.654)	**< 0.001**	0.682 (0.571-0.813)	**< 0.001**
Extremities	0.833 (0.783-0.886)	**< 0.001**	0.687 (0.635-0.744)	**< 0.001**	0.732 (0.634-0.844)	**< 0.001**
Trunk	0.553 (0.503-0.608)	**< 0.001**	0.569 (0.516-0.627)	**< 0.001**	0.626 (0.516-0.758)	**< 0.001**
Fat mass percent (%)						
Total	1.257 (1.214-1.300)	**< 0.001**	1.240 (1.198-1.283)	**< 0.001**	1.235 (1.144-1.333)	**< 0.001**
Upper limb	4.561 (3.646-5.704)	**< 0.001**	4.101 (3.275-5.134)	**< 0.001**	3.104 (2.023-4.764)	**< 0.001**
Lower limb	1.964 (1.762-2.188)	**< 0.001**	1.990 (1.773-2.232)	**< 0.001**	1.794 (1.425-2.257)	**< 0.001**
Extremities	1.647 (1.526-1.778)	**< 0.001**	1.615 (1.494-1.746)	**< 0.001**	1.499 (1.285-1.749)	**< 0.001**
Trunk	1.452 (1.372-1.537)	**< 0.001**	1.424 (1.345-1.508)	**< 0.001**	1.506 (1.299-1.744)	**< 0.001**

Model 1: unadjusted; Model 2: Model 1 additionally adjusted for age and sex; Model 3: Model 2 additionally adjusted for prediabetes or diabetes, overweight, central obesity, elevated BP, elevated triglycerides, low HDL, high triglyceride/HDL ratio. Bold values indicate if *P* < 0.05.

MAFLD, metabolic dysfunction-associated fatty liver disease; OR, odds ratio; CI, confidence interval.

Sensitivity and subgroup analyses were conducted for sex and weight. When stratified by sex (**Table 3**), similar results and trends were observed for both men and women. Conversely, no significant association was found in the normal-weight subgroup (**Table 4**).

**Table 3:** Associations between regional muscle mass (%)/fat mass (%) and MAFLD stratified by sex.

Variables	Male		Female	
	OR (95% CI)	*P*	OR (95% CI)	*P*
Muscle mass percent (%)				
Total	1.227 (1.179-1.277)	**< 0.001**	1.277 (1.193-1.367)	**< 0.001**
Upper limb	0.739 (0.549-0.994)	**0.046**	0.974 (0.581-1.632)	0.920
Lower limb	0.588 (0.521-0.663)	**< 0.001**	0.593 (0.488-0.719)	**< 0.001**
Extremities	0.681 (0.619-0.748)	**< 0.001**	0.698 (0.598-0.815)	**< 0.001**
Trunk	0.577 (0.514-0.647)	**< 0.001**	0.534 (0.441-0.646)	**< 0.001**
Fat mass percent (%)				
Total	0.692 (0.642-0.745)	**< 0.001**	0.662 (0.585-0.748)	**< 0.001**
Upper limb	3.844 (2.964-4.987)	**< 0.001**	4.993 (3.190-7.816)	**< 0.001**
Lower limb	1.939 (1.695-2.218)	**< 0.001**	2.145 (1.717-2.679)	**< 0.001**
Extremities	1.584 (1.446-1.735)	**< 0.001**	1.709 (1.468-1.990)	**< 0.001**
Trunk	1.392 (1.305-1.486)	**< 0.001**	1.518 (1.349-1.708)	**< 0.001**

The covariate was age; Bold values indicate if *P <*0.05.

MAFLD, metabolic dysfunction-associated fatty liver disease; OR, odds ratio; CI, confidence interval.

**Table 4:** Associations between regional muscle mass (%)/fat mass (%) and MAFLD stratified by weight.

Variables	Normal		Overweight/obesity	
	OR (95% CI)	*P*	OR (95% CI)	*P*
Muscle mass percent (%)				
Total	0.826 (0.620-1.100)	0.191	0.768 (0.710-0.830)	**< 0.001**
Upper limb	0.471 (0.159-1.398)	0.175	0.582 (0.411-0.824)	**0.002**
Lower limb	0.755 (0.510-1.118)	0.161	0.704 (0.623-0.797)	**< 0.001**
Extremities	0.791 (0.582-1.076)	0.136	0.766 (0.695-0.846)	**< 0.001**
Trunk	0.710 (0.497-1.015)	0.061	0.687 (0.612-0.772)	**< 0.001**
Fat mass percent (%)				
Total	1.129 (0.939-1.357)	0.198	1.183 (1.131-1.236)	**< 0.001**
Upper limb	2.706 (0.713-1.273)	0.143	2.654 (2.051-3.433)	**< 0.001**
Lower limb	1.431(0.775-2.643)	0.252	1.583 (1.375-1.823)	**< 0.001**
Extremities	1.336 (0.867-2.059)	0.189	1.392 (1.267-1.529)	**< 0.001**
Trunk	1.171 (0.871-1.575)	0.296	1.346 (1.246-1.455)	**< 0.001**

The covariate was age, sex; Bold values indicate if *P* < 0.05.

MAFLD, metabolic dysfunction-associated fatty liver disease; OR, odds ratio; CI, confidence interval.

### 3.3 Relationship between physical activity habits and MAFLD

**Figure 4:**
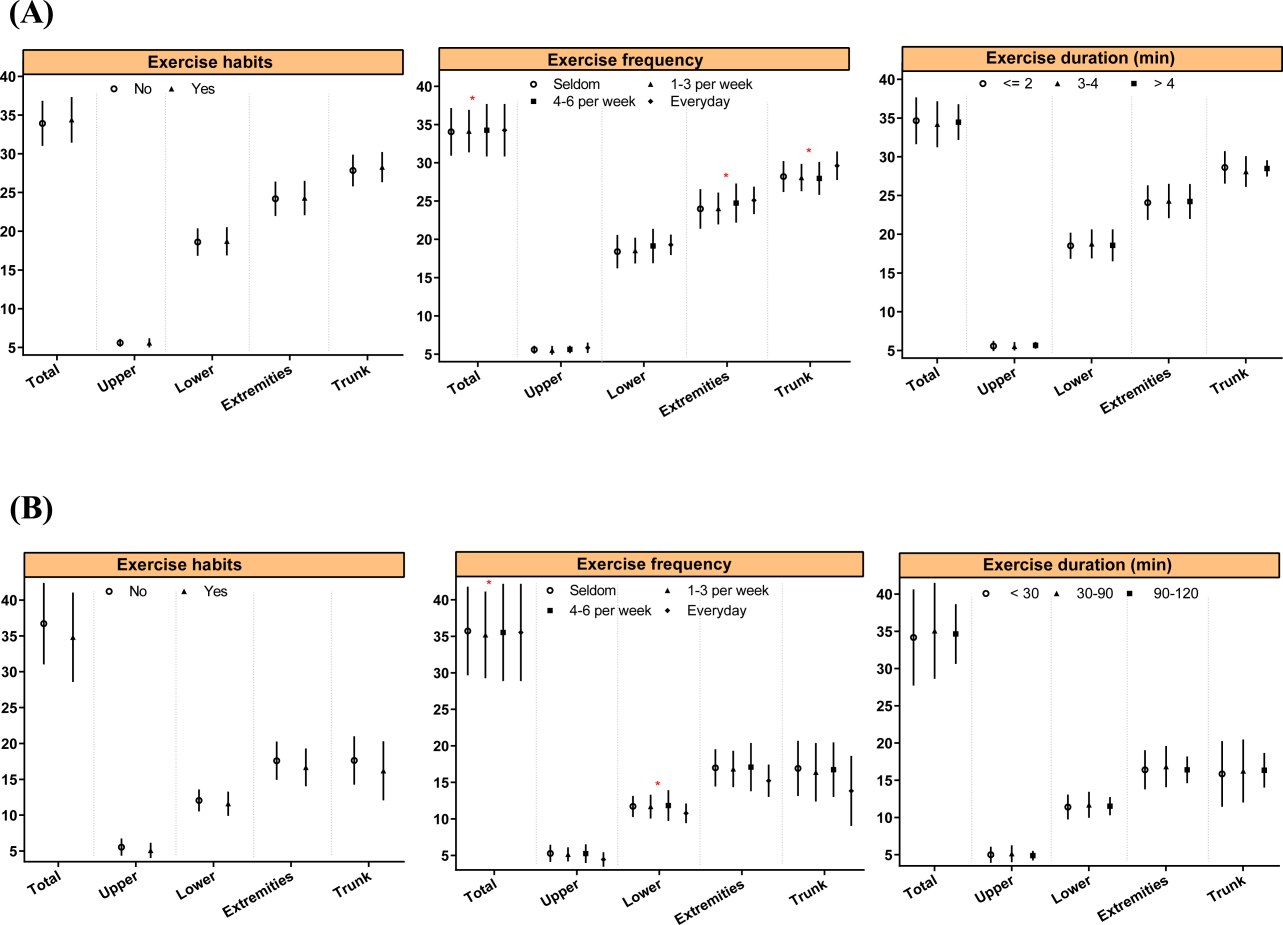
Associations between physical activity habits and body composition in MAFLD children. * indicate *P* < 0.05.

Regarding the relationship between physical activity habits and MAFLD, although the difference was not statistically significant, a greater proportion of individuals in the MAFLD group reported no exercise habits (14.93% *vs.* 10.17%). Similarly, the MAFLD group had a higher proportion of individuals with a lower frequency of exercise (15.32% *vs.* 11.07%) (**Table 1**). The associations between physical activity habits and body composition in children with MAFLD are presented in **Figure 4**. Compared to individuals who rarely exercise, those who engage in daily exercise had a greater overall muscle percentage (34.05 ± 3.11% *vs.* 34.26 ± 3.43%, *P* = 0.029), extremity muscle percentage (23.98 ± 2.58% *vs.* 25.1 ± 1.79%, *P* = 0.049), trunk muscle percentage (28.21 ± 2.03% *vs.* 29.62 ± 1.86%, *P* = 0.022), overall fat percentage (35.72 ± 6.06% *vs.* 35.53 ± 6.66%, *P* = 0.027), and lower limb fat percentage (11.71 ± 1.45% *vs.* 10.77 ± 1.33%, *P* = 0.040).

## 4 Discussion

In this study, we evaluated the relationship between segmental body composition and MAFLD in children. Our analysis revealed an inverse association between muscle mass and MAFLD, as well as an independent positive association with fat mass, especially with the muscle mass in the extremities and the fat mass in the upper limbs. Previous studies have shown that puberty has a significant impact on the prevalence of MAFLD across genders, with the prevalence in boys increasing as puberty progresses, while in girls, it peaks during the early stages of puberty (24). However, this study did not observe any gender differences, likely because the participants had not yet entered puberty. These findings underscore the significance of incorporating segmental body composition in assessing MAFLD risk.

MAFLD, formerly known as non-alcoholic fatty liver disease (NAFLD), underwent a nomenclature change in 2020 due to several limitations of the term “NAFLD” (25). These include potential confusion caused by the term “non-alcoholic”, its inability to account for adolescent alcohol misuse, and its failure to accurately reflect the underlying pathophysiology of metabolic dysfunction driven by insulin resistance. In 2023, the term was further updated to metabolic dysfunction-associated steatotic liver disease (MASLD) due to the potentially stigmatizing nature of the word “fatty” (26). Over the past few years, research have confirmed the link between low skeletal muscle mass and hepatic steatosis in both adults and children (27–34). For instance, Yodoshi et al. (35) showed that lower muscle mass was associated with a greater steatosis score in children (OR: 0.73, 95% CI: 0.56–0.96). A cross-sectional study of biopsy-confirmed NAFLD patients revealed that the lowest tertiles of relative muscle mass at baseline were correlated with an increased risk of developing NAFLD in children (OR: 2.80; 95% CI: 1.57–5.02) (36). In adults, Du et al. (37) found that greater lower limb, extremity and trunk muscle mass reduced the risk of NAFLD. However, research on the relationship between regional skeletal muscle mass and MAFLD in children is limited. Our findings demonstrated an inverse association between muscle mass across various regions and the occurrence of MAFLD, with the most significant effect observed in the muscle mass percentage in the extremities and lower limbs.

The pathogenic mechanisms underlying the negative correlation between muscle mass and hepatic steatosis involve multiple aspects. First, insulin resistance is strongly linked to the accumulation of ectopic fat in the liver. Skeletal muscle, the most efficient target organ for insulin-mediated glucose disposal in the body, is closely associated with insulin resistance (38, 39). Reduced muscle mass is also associated with a lower basal metabolic rate, which further exacerbates insulin resistance (8). In addition, chronic inflammation and enhanced oxidative stress are closely associated with muscle mass and the occurrence and progression of MAFLD (40). Lastly, skeletal muscle, which acts as an endocrine organ, plays a crucial role by secreting myokines, such as interleukin-6 and irisin, to regulate metabolic processes. These myokines are involved in the development of MAFLD (41). However, the relationship between the regional distribution of muscle mass and MAFLD remains unclear. One possible explanation is that the association between lower limb muscle mass and insulin resistance may be stronger than the association between upper limb muscle mass and insulin resistance.

Similarly, the role of body fat distribution in the development of MAFLD has garnered significant attention in the field of hepatology. Numerous studies have consistently highlighted the crucial role of abdominal fat deposition in the development of MAFLD (42, 43). Ciardullo et al. revealed a remarkable correlation between the android/gynoid ratio and hepatic steatosis (14). A significant correlation has also been found between a higher android-to-gynoid fat ratio and an increased risk of MAFLD in the Danish pediatric population (44). Our study confirmed the association between abdominal fat percentage and pediatric MAFLD and revealed for the first time that the correlation between upper limb fat percentage and pediatric MAFLD is even more significant. Recent studies exploring the relationship between upper limb fat and fatty liver have primarily been conducted in adults. Zhang et al. reported a positive correlation between adiposity levels in the upper limbs (measured using triceps and biceps skinfold thicknesses) and histological liver damage in patients with MAFLD (45). Studies have shown that upper limb obesity is a significant risk factor for various metabolic disorders (46). In metabolically unhealthy individuals with normal weight, fat tends to accumulate preferentially in the upper body (subcutaneous and visceral fat in the abdomen) and liver (47). As the liver plays an important role in the metabolism of carbohydrates and lipids (48), we hypothesize that the concentration of free fatty acids (FFA) in the portal vein after lipolysis may be significantly higher than in the arterial circulation. Thus, the liver may be exposed to higher concentrations of FFAs, which could increase the risk of developing MAFLD.

In patients with chronic liver disease, abnormal autophagy can impair muscle synthesis and increase protein degradation (49). Physical exercise can rescue impaired mTORC1 signaling by stimulating phosphatidic acid, thereby maintaining muscle mass through protein synthesis activation and autophagy inhibition (50). In particular, resistance exercise has been shown to effectively stimulate skeletal muscle protein synthesis, contributing to the treatment of chronic liver disease (51). Compared to diets high in unsaturated fats, those rich in saturated fats resulted in elevated intrahepatic triglyceride levels, which exacerbated hepatic lipid accumulation and impaired metabolic function (52). Therefore, for patients with MAFLD, high-quality diets, such as the Mediterranean diet, have become a crucial therapeutic approach. Our study aims to investigate regional body composition in relation to CAP-confirmed MAFLD in children (53). This approach provides a more accurate and comprehensive analysis of the association between these two factors, overcoming the limitations of previous studies that primarily focused on abdominal and overall body composition. This study holds clinical significance because it supports the use of body composition measurements obtained via BIA to assess the risk of MAFLD in adolescents. Moreover, our study provides a theoretical basis for developing treatment strategies to reduce the risk of MAFLD based on the association between regional body composition and MAFLD. For instance, protein supplementation (54) and resistance exercise (55) can increase muscle mass, particularly in the limbs, and reduce body fat, especially in the upper limbs and abdominal region, thus lowering the risk of MAFLD.

However, this study has several limitations. First, the study utilizes a cross-sectional design, meaning that data were collected at a single point in time. This design restricts our ability to make causal inferences, as we cannot determine the directionality or temporal sequence of the relationships between variables. To address this limitation, future research should consider longitudinal designs to better explore causal relationships. Additionally, incorporating experimental or randomized controlled trial methodologies could further validate our findings. Second, the potential selection bias arising from the specific recruitment process, including the inclusion of participants with chronic diseases and those using certain medications, should be acknowledged as a limitation of this study. Furthermore, we used BIA, a noninvasive and straightforward method, to assess body composition. The BIA method is widely used due to its rapid, simple, cost-effective, non-invasive, and safe characteristics. However, the accuracy of measurements obtained through BIA can be influenced by factors such as the BIA device, the subject’s body composition, hydration status, and health conditions, leading to potential measurement error. Therefore, further research is needed to explore the relationship between body composition and MAFLD using more accurate measurements, such as densitometry. Moreover, when selecting variables for inclusion, some may have been omitted or missing, which could introduce potential confounders that weren’t accounted for in the models. Finally, while CAP has been proven to be an effective diagnostic tool for detecting liver fat, it might be affected by factors, such as skin-to-capsule distance, age, or intercostal space width.

## 5 Conclusion

This study demonstrates that lower regional muscle percentages, particularly in the extremities, and higher regional fat percentages, especially in the upper limbs, are significantly associated with an increased risk of MAFLD in prepubertal children. Therefore, clinicians should incorporate segmental body composition analysis into routine risk assessments for pediatric MAFLD, with a particular focus on limb muscle mass and upper limb fat distribution. Moreover, personalized exercise interventions aimed at reducing upper limb fat while preserving muscle mass may represent an effective approach for managing MAFLD in children. Further studies are needed to validate these findings and explore stratified intervention strategies based on specific body composition characteristics, thereby providing more precise guidance for clinical practice.

## Data Availability

The raw data supporting the conclusions of this article will be made available by the authors, without undue reservation.
